# Clinical characteristics of systemic sclerosis patients with occupational silicosis

**DOI:** 10.1007/s10067-023-06706-5

**Published:** 2023-09-15

**Authors:** Xiaocong Huo, Zhiyu Zeng, Yongjun Lin, Jinying Lin, Dong Xu

**Affiliations:** 1 Department of Cardiology, The First Affiliated Hospital of Guangxi Medical University, Guangxi Key Laboratory of Precision Medicine in Cardio-cerebrovascular Diseases Control and Prevention, Guangxi Clinical Research Center for Cardio-cerebrovascular Disease. The People’s Hospital of Guangxi Zhuang Autonomous Region, Guangxi Academy of Medical Sciences, No. 6 Shuangyong Road, Nanning, Guangxi 530021 China; 2 Department of Cardiology, The First Affiliated Hospital of Guangxi Medical University, Guangxi Key Laboratory of Precision Medicine in Cardio-Cerebrovascular Diseases Control and Prevention, Guangxi Clinical Research Center for Cardio-Cerebrovascular Diseases, Nanning, Guangxi 530021 China; 3 Guangxi Workers’ Hospital, Nanning, 530021 Guangxi China; 4 The People’s Hospital of Guangxi Zhuang Autonomous Region, Guangxi Academy of Medical Sciences, Nanning, 530021 Guangxi China; 5 Peking Union Medical College Hospital, Chinese Academy of Medical Sciences & Peking Union Medical College, Beijing, 100032 China

**Keywords:** Cardiac involvement, Propensity score matching, Silicosis, Systemic sclerosis

## Abstract

To explore the clinical characteristics of systemic sclerosis complicated with silicosis. The systemic sclerosis patients treated in the Guangxi Workers’ Hospital and the People's Hospital of Guangxi Zhuang Autonomous Region from January 2000 to December 2020 were divided into the systemic sclerosis with silicosis group and the systemic sclerosis without silicosis group. Survival analysis was performed using Kaplan–Meier estimates the Cox proportional hazards model. A propensity score matching was applied in order to avoid the selection bias.

Over the past 20 years, 72 systemic sclerosis patients with silicosis and 238 systemic sclerosis patients without silicosis were treated in the two hospitals. The systemic sclerosis patients with silicosis group had more males (*P* < 0.000),lower mean age at onset of SSc (*P* < 0.000), more frequent occurrence of weight loss (*P* = 0.028), smoking (*P* < 0.000), tuberculosis (*P* < 0.000), cardiac involvement (*P* < 0.000), ILD (*P* = 0.017), pulmonary hypertension (*P* = 0.024), elevated BNP (*P* < 0.000). With regards to the multivariate Cox regression analysis, silicosis was related with a higher overall mortality before (HR = 3.666, 95% CI = 1.440–11.234, *p* = 0.025) and after the propensity score matching analysis (HR = 2.817, 95% CI = 1.196–10.764, *p* = 0.014). Independent risk factors for overall mortality were Gangrene (HR = 3.003, 95% CI = 1.343–9.431), Cardiac involved (HR = 5.370, 95% CI = 1.910–15.472), Scl-70 (HR = 3.569, 95% CI = 1.333–10.869), Elevated BNP (HR = 2.135, 95% CI = 1.293–9.564).

Concomitant silicosis worsens systemic sclerosis patients’ prognoses. Gangrene, Scl-70, elevated BNP and cardiac involvement are independent risk factors for overall mortality.

**Table:** 

**Key Points** •*Concomitant silicosis worsens SSc patients’ prognoses.* •*For individuals with occupational exposure, close observation of the symptoms of SSc, early diagnosis, and interruption of exposure may improve the prognosis.* •*Gangrene, Scl-70, elevated BNP and cardiac involvement are independent risk factors for overall mortality. *

**Key Points**

•*Concomitant silicosis worsens SSc patients’ prognoses.*

•*For individuals with occupational exposure, close observation of the symptoms of SSc, early diagnosis, and interruption of exposure may improve the prognosis.*

•*Gangrene, Scl-70, elevated BNP and cardiac involvement are independent risk factors for overall mortality.
*

## Introduction

Systemic sclerosis (SSc) is an immune-mediated rheumatic disease characterized by fibrosis of skin, internal organs, and vasculopathy. Although the etiology of this complex and heterogeneous condition remains an enigma, SSc has high morbidity and mortality [1]. The pathogenesis of SSc involves immune, inflammatory, vascular, and fibrotic pathways [1].

Increasing evidence shows that SSc is related to various occupational and environmental factors, such as silica exposure [2–4]. The risk of SSc in silicosis patients is 28 times higher than that in the general population [5]. The clinical characteristics of SSc patients with silica exposure are different from those of SSc patients without silica exposure, such as the degree of dermatological disease and of visceral involvement, and the degree and type of autoantibodies [6, 7].

The mechanism by which silica exposure leads to systemic autoimmune disease remains unknown, while there are few studies on silica exposure and silicosis that may help identify immune processes that precede autoimmune development. Crystalline silica may induce T-cell and B-cell proliferation by activating T-cell and BCR complexes [8]. In the presence of macrophages, silica particles can trigger an inflammatory response, which stimulates fibroblasts to proliferate and produce collagen [9].

At present, there are few data regarding SSc patients with silicosis. We have collected the clinical and prognostic data of SSc patients with silicosis in two hospitals in Guangxi, in order to improve clinicians' understanding of the disease.

## Methods

### Patients and study design

SSc patients were retrospectively collected from January 2000 to December 2020 in the Guangxi Workers’ Hospital and the People's Hospital of Guangxi Zhuang Autonomous Region. All patients met the 1980 American College of Rheumatology (ACR) SSc classification criteria [10]. According to Leroy's definition, those patients were classified as diffuse cutaneous (dc-SSc) and limited cutaneous SSc (lc-SSc) [11]. The Ethics Committee of Guangxi Workers’ Hospital approved this study (NO:2,019,001). Informed consent was obtained from participants.

Silicosis was diagnosed according to the guidelines of the ILO International classification of radiographs of pneumoconiosis [12] by the silicosis diagnosis and treatment group of Guangxi Workers’ Hospital based on the results of chest X-ray, computed tomography (CT), and dust exposure history. According to Kelly's Textbook of Rheumatology [13], muscle involvement is defined as tmyalgia, muscle weakness, or elevated creatine kinase. Cardiac involvement is defined asarrhythmia on electrocardiogram (ECG), left ventricular ejection fraction (LVEF) ≤ 45%, pericardial effusion, elevated troponin, or valve regurgitation. Myocardial involvement is defined as low LVEF(≤ 45%) or elevated troponin(> 0.1 ng/ml). Gastrointestinal involvement is defined as gastroesophageal reflux disease, dysphagia, vomit, early satiation, bloating, constipation, diarrhea. Renal involvement is defined as scleroderma renal crisis (SRC), serum creatinine ≥ 1.5 mg/dL (132.6 µmol/L), microscopic hematuria, or 24-h urinary protein ≥ 0.5 g/24 h. Pulmonary arterial hypertension (PH) is defined as a mean pulmonary arterial pressure of > 25 mmHg at rest or > 30 mmHg during exercise together with a pulmonary capillary wedge pressure of < 15 mmHg through right heart catheterisation or a pulmonary artery systolic pressure > 40 mmHg at rest based on an echocardiogram test. Pulmonary tuberculosis (TB) was diagnosed according to the guidelines of Official American Thoracic Society/Infectious Diseases Society of America/Centers for Tuberculosis [14], by the Multidisciplinary diagnosis of pulmonologists, physicians in infectious department, and radiologist. Interstitial lung disease (ILD) was defined as ground glass opacification or fibrosis on high-resolution computed tomography (HRCT).

Irreversible organ injury included chronic respiratory failure, chronic cardiac failure, and limb gangrene, chronic kidney disease.

### Statistical analysis

The SPSS 22.0 statistical software package was used for statistical analysis. Continuous variables are expressed as means ± SDs or medians and interquartile ranges. Categorical parameters are expressed as incidences and percentages. Independent sample t-test was used for continuous variables, Wilcoxon signed-rank test was used for continuous variables with non-normal distribution, and chi-squared test or Fisher’s exact test were used for categorical variables. Survival analysis was carried out using the Kaplan–Meier method and was compared to the log-rank test for equality of survival curves. Multivariate analyses were also performed using the Cox proportional hazards model to identify independent prognostic factors. Furthermore, we used propensity score matching(PSM) in estimation the effects of silicosis on outcome variables, to avoid the selection bias. All statistical tests were two-tailed, and P values less than 0.05 were considered significant.

## Results

In total, 310 patients with SSc were diagnosed from January 2000 to December 2020 in the Guangxi Workers’ Hospital and the People's Hospital of Guangxi Zhuang Autonomous Region. 238 SSc patients (76.8%) without silicosis had no history of dust exposure. There were 72 cases (23.2%) of SSc with silicosis. The 72 workers included 21 blasting workers, 16 manganese miners, 11 gold miners, 8 coal miners, 4 antimony miners, 3 tile cutters, 2 gem cutters, 2 tin miners, 2 zinc miners, 2 cement and brick factory workers, and 1 stone factory worker. Eight out of 72 cases (11.1%) of SSc with silicosis had no respiratory symptoms and were diagnosed during the physical examination for occupational diseases. Among the remaining 64 patients(88.9%), the median time from dust exposure to silicosis symptoms, including cough and dyspnea, was 17.01 (interquartile range:7.70–23.77) years, ranging from 1.00 to 45.03 years. The median time from dust exposure to the first SSc-related symptom other than Raynaud’s phenomenon, including skin sclerosis, fingertip ulcers, joint swelling, myalgia, cough and shortness of breath, swallowing obstruction, was 18.90 (interquartile range:11.17–24.02) years, ranging from 2.00 to 47.03 years. Twenty-one cases out of 72 cases (29.2%) of SSc with silicosis SSc symptoms developed before silicosis did, over 1.00 (interquartile range:0.30–2.09) years, ranging from 0.30 to 7.02 years. Forty-seven cases out of 72 cases (65.3%) of SSc with silicosis, silicosis developed before SSc symptoms, over 3.00 (interquartile range:1.84–6.93) years, ranging from 0.08 to 14.84 years. Four patients(5.6%) presented with simultaneous SSc symptoms and silicosis (Table 1).

**Table 1:** Comparison of clinical manifestations and laboratory data between the two groups

	Cohort(n = 310)					Propensity score matching(n = 101)				
	Total	SSc with silicosis	SSc without silicosis	p-value	*t/χ*^2^*/Z*	Total	SSc with silicosis	SSc without silicosis	p-value	*t/χ*^2^*/Z*
Number of patients	310	72	238	-	-	101	45	56	-	-
Gender (F)	165[53.2%]	1[1.4%]	164[68.9%]	0.000	101.220	3[3.0%]	1[2.2%]	2[3.6%]	1.000	-
Age at onset of SSc (years)		47.83 ± 7.97	55.42 ± 11.09	0.000	6.412		50.00 ± 7.66	50.70 ± 8.43	0.488	0.430
Dust exposure history (years)		5.0 (3.0–8.8)	-	-	-		4.0 (2.8–8.5)	-	-	-
Silicosis stage										
Phase I	42	42[58%]	-	-	-	26	26[57.8%]	-	-	-
Phase II	25	25[35%]	-	-	-	18	18[40.0%]	-	-	-
Phase III	5	5[7%]	-	-	-	2	2[4.4%]	-	-	-
Tuberculosis	50[16.1%]	43[59.7%]	7[2.9%]	0.000	131.745	28[27.7%]	27[60.0%]	1[1.8%]	0.000	42.199
Smoking	57[18.4%]	25[34.7%]	32[13.4%]	0.000	16.676	45[44.6%]	18[40.0%]	27[48.2%]	0.409	0.681
Weight loss	81[26.1%]	26[36.1%]	55[23.1%]	0.028	4.841	31[30.7%]	16[35.6%]	15[26.8%]	0.342	0.902
Classification										
dc-SSc	257[82.9%]	63[87.5%]	194[81.5%]	0.237	1.398	83[82.2%]	40[88.9%]	43[76.8%]	0.114	2.496
lc-SSc	53[17.1%]	9[12.5%]	44[18.5%]			18[17.8%]	5[11.1%]	13[23.2%]		
Overlapping syndrome	14[4.5%]	0[0.0%]	14[5.9%]	0.046	-	1[1.0%]	0[0.0%]	1[1.8%]	1.000	-
mRSS		16 (9.25–26)	12 (8–17)	0.291	1.056		16.0 (10.0–25.0)	12.0 (8.0–18.0)	0.083	1.734
Raynaud’s phenomenon	265[85.5%]	54[75.0%]	211[88.7%]	0.004	8.307	88[87.1%]	36[80.0%]	52[92.9%]	0.055	3.678
Arthritis/arthralgia	94[30.3%]	14[19.4%]	80[33.6%]	0.022	5.253	29[28.7%]	9[20.0%]	20[35.7%]	0.083	3.010
Fingertip ulcers	89[28.7%]	20[27.8%]	69[29.0%]	0.842	0.040	35[34.7%]	11[24.4%]	24[42.9%]	0.053	3.735
Gangrene	34[11.0%]	10[13.9%]	24[10.1%]	0.365	0.820	10[9.9%]	5[11.1%]	5[8.9%]	0.748	-
Subcutaneous calcinosis	13[4.2%]	3[4.2%]	13[4.2%]	0.990	0.000	4[4.0%]	3[6.7%]	1[1.8%]	0.211	1.563
Telangiectasia	37[11.9%]	2[2.8%]	35[14.7%]	0.006	7.483	12[11.9%]	1[2.2%]	11[19.6%]	0.007	7.232
Myositis	86[27.7%]	18[25.0%]	68[28.6%]	0.553	0.352	27[26.7%]	10[22.2%]	17[30.4%]	0.359	0.843
Renal involved	15[4.8%]	1[1.4%]	14[5.9%]	0.206	-	3[3.0%]	0[0.0%]	3[5.4%]	0.251	-
Cardiac involved	70[22.6%]	33[45.8%]	37[15.5%]	0.000	29.005	35[34.7%]	22[48.9%]	13[23.2%]	0.007	7.263
Pericardial effusion	43[13.9%]	16[22.2%]	27[11.3%]	0.019	5.475	18[17.8%]	13[28.9%]	5[8.9%]	0.009	6.787
Myocardial involvement	25[8.1%]	10[13.9%]	15[6.3%]	0.038	4.291	11[10.9%]	8[17.8%]	3[5.4%]	0.058	-
Tricuspid regurgitation	33[10.6%]	29[40.3%]	4[1.7%]	0.000	86.574	23[22.8%]	22[48.9%]	1[1.8%]	0.000	31.477
Coronary artery involvement	2[0.6%]	0[0.0%]	2[0.8%]	1.000	-	0[0.0%]	0[0.0%]	0[0.0%]	-	-
Gastrointestinal involved	92[29.7%]	21[29.2%]	71[29.8%]	0.914	0.012	34[33.7%]	12[26.7%]	22[39.3%]	0.182	1.779
ILD	224[72.3%]	60[83.3%]	164[68.9%]	0.017	5.739	78[77.2%]	39[86.7%]	39[69.6%]	0.043	4.112
Velcro crackles	94[30.3%]	38[52.8%]	56[23.5%]	0.000	22.382	46[45.5%]	25[55.6%]	21[37.5%]	0.070	3.280
PH	69[22.3%]	23[31.9%]	46[19.3%]	0.024	5.085	31[30.7%]	15[33.3%]	16[28.6%]	0.606	0.266
Hemoglobin(g/L)		128.92 ± 18.06	115.39 ± 20.52	0.759	5.391		128.82 ± 18.67	118.95 ± 22.96	0.534	2.331
ANA	297[95.8%]	62[86.1%]	235[98.7%]	0.000	-	97[96.0%]	41[91.1%]	56[100.0%]	0.036	-
Scl-70	247[79.7%]	57[79.2%]	190[79.8%]	0.902	0.015	86[85.1%]	39[86.7%]	47[83.9%]	0.701	0.148
ACA	30[9.7%]	6[8.3%]	24[10.1%]	0.660	0.194	8[7.9%]	4[8.9%]	4[7.1%]	1.000	-
Elevated Creatine kinase	92[29.7%]	22[30.6%]	70[29.4%]	0.852	0.035	31[30.7%]	13[28.9%]	18[32.1%]	0.725	0.124
Elevated Myoglobin	65[21.0%]	4[5.6%]	61[25.6%]	0.000	13.443	18[17.8%]	3[6.7%]	15[26.8%]	0.009	6.896
Elevated BNP	98[31.6%]	41[56.9%]	57[23.9%]	0.000	27.836	49[48.5%]	28[62.2%]	21[37.5%]	0.013	6.105
Positive Coombs test	23[7.4%]	1[1.4%]	23[7.4%]	0.026	4.965	6[5.9%]	1[2.2%]	5[8.9%]	0.222	-
Elevated Cr	26[8.4%]	4[5.6%]	22[9.2%]	0.323	0.979	11[10.9%]	2[4.4%]	9[16.1%]	0.062	3.476
Elevated BUN	32[10.3%]	6[8.3%]	26[10.9%]	0.527	0.401	12[11.9%]	3[6.7%]	9[16.1%]	0.147	2.108
Elevated IgG	81[26.1%]	21[29.2%]	60[25.2%]	0.503	0.448	31[30.7%]	15[33.3%]	16[28.6%]	0.606	0.266
Decreased PaO2	24[7.7%]	12[16.7%]	12[5.0%]	0.001	10.458	12[11.9%]	7[15.6%]	5[8.9%]	0.306	1.047
Elevated CRP	70[22.6%]	26[36.1%]	44[18.5%]	0.002	9.821	22[21.8%]	12[26.7%]	10[17.9%]	0.286	1.137
Elevated ESR	154[49.7%]	52[72.2%]	102[42.9%]	0.000	19.067	54[53.5%]	31[58.9%]	23[41.1%]	0.005	7.760
Elevated RF	24[7.7%]	2[2.8%]	22[9.2%]	0.072	3.236	7[6.9%]	2[4.4%]	5[8.9%]	0.457	-

SSc: systemic sclerosis; dc: diffuse cutaneous; lc: localized cutaneous; mRSS: modified Rodnan skin score; ILD: interstitial lung disease; PH: pulmonary hypertension; ANA: antinuclear antibodies; ACA: anti-centromere antibodies; BUN: blood urea nitrogen; IgG: immunoglobulin G; PaO2: partial pressure of oxygen; CRP: C-reactive protein; ESR: erythrocyte sedimentation rate

### Clinical manifestations

The SSc with silicosis group had more males (P = 0.000) and a lower mean age at onset of SSc (P = 0.000). The SSc patients with silicosis had morefrequent occurrence of weight loss(P = 0.028) (Weight loss was defined as unintentional weight loss of 5% or more over the previous year according to Fried’s criterion [15]), smoking (P = 0.000), cardiac involved (P = 0.000), ILD (P = 0.017), velcro crackles (P = 0.000), PH (P = 0.024), elevated BNP (P = 0.000), decreased PaO2 (P = 0.001), elevated CRP (P = 0.002), elevated ESR (P = 0.000), while the incidence of Overlapping syndrome(P = 0.046), Raynaud’s phenomenon(P = 0.004), arthritis/arthralgia(P = 0.022), telangiectasia (P = 0.006), elevated myoglobin (P = 0.000) were low (Table 1).

To reduce bias from confounding factors between groups, PSM was performed with a tolerance of 0.05. Matching covariates consisted of gender, age. After matching, 45 cases in the SSc with silicosis group and 56 cases in the SSc without silicosis group were obtained. The SSc patients with silicosis had morefrequent occurrence of TB (P = 0.000), cardiac involved (P = 0.007), ILD (P = 0.043), elevated BNP (P = 0.013), elevated ESR (P = 0.005), while the incidence of telangiectasia (P = 0.007), elevated myoglobin (P = 0.009) were low (Table 1).

The SSc with silicosis group had more cases of TB (P = 0.000). The TB type classification was either pulmonary TB or extra-pulmonary TB. A total of 50 patients with TB were diagnosed in our study, mycobacterium TB was found in 11 cases (22.92%) by bronchoscopy lavage and in nine cases (18.75%) by sputum smear, and the remaining patients (58.33%) were diagnosed by pulmonary imaging and examination of clinical manifestations.

### Treatment

The patients in both groups were given hormonal and immunosuppressive therapy according to their condition, while those with silicosis were given pulmonary lavage, oxygen therapy, antifibrosis treatment, and anti-infective treatment if necessary. SSc patients without silicosis were more likely to be treated with high dose hormone impulsion therapy(≥ 250 mg/d of prednisone or equivalent doses of methylprednisolone, dexamethasone or hydrocortisone) (P = 0.000) and adequate hormone therapy (> 30–100 mg/d of prednisone)(P = 0.023), while the SSc patients with silicosis were more likely to be treated with moderate hormone therapy(> 7.5–30 mg/d of prednisone) (P = 0.000). There was no statistical difference between the two groups using low dose hormone therapy(≤ 7.5 mg/d of prednisone)(P = 0.140). Most of the 22 SSc patients without silicosis were treated with high dose hormones before 2015 (18 cases, 82%). The reasons for the use included short course of disease (12 cases, 55%), rapid progression of skin sclerosis in a short period of time (7 cases, 32%), and short-term progression of ILD (9 cases, 41%). In recent years, the use of high dose hormones in SSc has been controversial because of its possible relationship with SRC.

Among the 22 SSc patients treated with high dose hormone impulsion therapy, 5 patients (22.7%) developed SRC, and the time from high dose hormone impulsion therapy to the occurrence of SRC was 0.5, 1,2,6,15 months, respectively. Two of them (6 months and 15 months) developed SRC due to disease recurrence, while the other three patients had short-term progressive interstitial pneumonia, significantly elevated muscle enzymes, and rapidly progressive skin sclerosis. It was uncertain whether the emergence of SRC was caused by the natural progression of the disease or the impact of high dose hormone.

In terms of the use of immunosuppressants, cyclophosphamide (CTX) (P = 0.000) was used more in the SSc patients without silicosis, and azathioprine (AZA) (P = 0.000) was used more in the SSc patients with silicosis. There was no statistical difference between the two groups using methotrexate (MTX) (P = 0.163) and cyclosporine (CSA) (P = 1.000) (Table 2).

**Table 2:** Comparison of therapy between the two groups

	Cohort(n = 310)					Propensity score matching(n = 101)				
	Total	SSc with silicosis	SSc without silicosis	p-value	*χ*^2^	Total	SSc with silicosis	SSc without silicosis	p-value	*χ*^2^
Hormone therapy										
High dose hormone impulsion therapy	22[7.1%]	0[0.0%]	22[9.2%]	0.007	7.164	8[7.9%]	0[0.0%]	8[14.3%]	0.008	-
Adequate hormone therapy	89[28.7%]	13[18.1%]	76[31.9%]	0.023	5.201	21[20.8%]	10[22.2%]	11[19.6%]	0.751	0.101
Moderate hormone therapy	104[33.5%]	42[58.3%]	62[26.1%]	0.000	25.841	52[51.5%]	26[57.8%]	26[46.4%]	0.257	1.287
Low-dose hormone therapy	95[30.6%]	17[23.6%]	78[32.8%]	0.140	2.183	20[19.8%]	9[20.0%]	11[19.6%]	0.964	0.002
Immunosuppressive therapy										
CTX	180[58.1%]	28[38.9%]	152[63.9%]	0.000	14.162	62[61.4%]	21[46.7%]	41[73.2%]	0.006	7.419
MTX	112[36.1%]	31[43.1%]	81[34.0%]	0.163	1.950	28[27.7%]	16[35.6%]	12[21.4%]	0.115	2.485
AZA	7[2.3%]	7[9.7%]	0[0.0%]	0.000	-	5[5.0%]	5[11.1%]	0[0.0%]	0.015	-
CSA	2[0.6%]	0[0.0%]	2[0.8%]	1.000	-	1[1.0%]	0[0.0%]	1[1.8%]	1.000	-
Others	9[2.9%]	6[8.3%]	3[1.3%]	0.006	-	5[5.0%]	3[6.7%]	2[3.6%]	0.654	-

High dose hormone impulsion therapy: ≥ 250 mg/d of prednisone or equivalent doses of methylprednisolone, dexamethasone or hydrocortisone; Adequate hormone therapy: > 30–100 mg/d of prednisone;

Moderate hormone therapy: > 7.5–30 mg/d of prednisone; Low-dose hormone therapy: ≤ 7.5 mg/d of prednisone

CTX: cyclophosphamide; MTX: methotrexate; AZA: azathioprine; CSA: cyclosporine

### Prognosis

During a follow-up of 44.0 (interquartile range:35.2–54.0) months in the SSc with silicosis group, 24 cases (33.3%) died, and 10 cases (13.9%) sustained irreversible organ injury. The causes of death included respiratory failure (12 cases, 16.7%), cardiac failure (12 cases, 16.7%), the causes of sustained irreversible organ injury included respiratory failure (4 cases, 5.6%), cardiac failure (5 cases, 6.9%), renal insufficiency and gangrene (1 case, 1.4%).

During a follow-up of 44.5 (interquartile range:17.0–86.1) months in the SSc without silicosis group, 12 cases (5.0%) died, and 30 cases (12.6%) sustained irreversible organ injury. The causes of death included respiratory failure (10 cases, 4.2%), cardiac failure (2 cases, 0.8%), the causes of sustained irreversible organ injury included respiratory failure (4 cases, 1.7%), cardiac failure (5 cases, 2.1%), renal failure (15 cases, 6.3%), and gangrene (16 cases, 6.7%) (Table 3).

**Table 3:** Comparison of Survival Between the two groups

	Cohort(n = 310)					Propensity score matching(n = 101)				
	Total	SSc with silicosis	SSc without silicosis	p-value	*χ*^2^	Total	SSc with silicosis	SSc without silicosis	p-value	*χ*^2^
Death	36[11.6%]	24[33.3%]	12[5.0%]	0.000	43.105	18[17.8%]	12[26.7%]	6[10.7%]	0.037	4.335
Death and irreversible organ injury	76[24.5%]	34[47.2%]	42[17.6%]	0.000	26.127	29[28.7%]	18[40.0%]	11[19.6%]	0.025	5.052

**Fig. 1:**
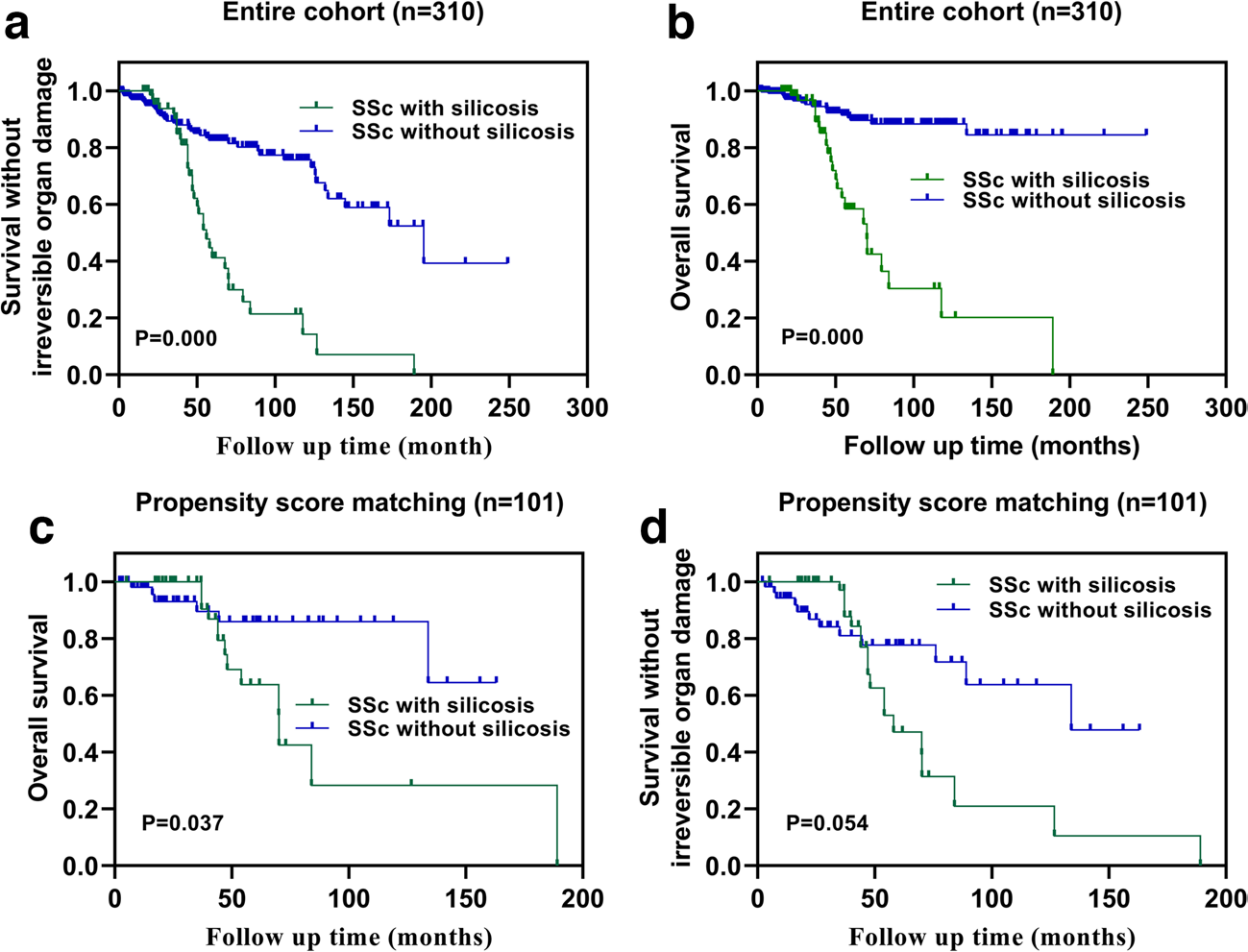
Note: This data is mandatory. Please provide

The median follow-up time was 44.0 (interquartile range:20.0–73.1) months. According to the Kaplan–Meier method, the SSc with silicosis group had higher overall mortality rate (P = 0.000, HR = 5.410, 95%CI = 2.532–11.560), higher incidence of death and irreversible organ damage (P = 0.002, HR = 3.408, 95%CI = 1.926–6.031) (Fig. 1a,b).

After PSM, 45 patients in the SSc with silicosis group and 56 patients in the SSc without silicosis group were included. The median follow-up time was 40.0(interquartile range:20.2–60.7) months. The SSc with silicosis group had a high overall mortality rate (P = 0.037, HR = 2.543, 95%CI = 1.032–6.269), and there was no statistical difference in the rates of death and irreversible organ damage (P = 0.054, HR = 1.948, 95%CI = 0.947–4.009) between the two groups (Fig. 1c,d).

Multivariate analyses were performed of the 310 patients. Overall mortality is the dependent variable, all factors with a p-value < 0.05 detected in univariate analyses were included in multivariate analyses. SSc with silicosis was related with a higher overall mortality (HR = 3.666, 95% CI = 1.440–11.234, p = 0.025), and the association was still seen after PSM (HR = 2.817, 95% CI = 1.196–10.764, p = 0.014).

In addition, other independent variables were fingertip ulcers (HR = 3.208, 95% CI = 1.149–10.986), gangrene (HR = 8.926, 95% CI = 2.408–25.965), cardiac involved (HR = 4.225, 95% CI = 1.090–16.383), velcro crackles (HR = 2.918, 95% CI = 1.340–6.354), Scl-70 (HR = 3.608, 95% CI = 1.189–10.368), elevated BNP (HR = 2.600, 95% CI = 1.070–8.776). After PSM, independent risk factors for all-cause mortality were gangrene (HR = 3.003, 95% CI = 1.343–9.431), cardiac involved (HR = 5.370, 95% CI = 1.910–15.472), Scl-70 (HR = 3.569, 95% CI = 1.333–10.869), elevated BNP (HR = 2.135, 95% CI = 1.293–9.564) (Table 4).

**Table 4:** Univariate and multivariate Cox regression analyses estimating the risk factors of OS in the cohort study and after propensity score matching

	Cohort(n = 310)			Propensity score matching (n = 101)		
	Univariate analysis	Multivariate analysis		Univariate analysis	Multivariate analysis	
	P value	HR (95% CI)	P value	P value	HR (95% CI)	P value
Group		3.666(1.440–11.234)	0.025		2.817(1.196–10.764)	0.014
Gender (F)	0.000	14.687(3.525–61.195)	0.000	0.521		
Age at onset of SSc (years)	0.006	2.315(0.558–6.257)	0.236	0.259		
Tuberculosis	0.000	1.158(0.388–3.457)	0.793	0.137		
Smoking	0.056			0.322		
Weight loss	0.001	1.175(0.444–3.110)	0.745	0.243		
Classification	0.692			0.949		
Overlapping syndrome	0.407			0.859		
mRSS	0.268			0.339		
Raynaud’s phenomenon	0.561			0.310		
Arthritis/arthralgia	0.819			0.753		
Fingertip ulcers	0.000	3.208(1.149–10.986)	0.036	0.448		
Gangrene	0.000	8.926(2.408–25.965)	0.004	0.009	3.003(1.343–9.431)	0.006
Subcutaneous calcinosis	0.000	1.760(0.320–9.690)	0.516	0.006	3.208(0.449–10.986)	0.136
Telangiectasia	1.000			0.325		
Myositis	0.005	1.744(0.514–5.914)	0.372	0.045	1.181(0.165–8.436)	0.868
Renal involved	0.000	1.982(0.261–15.053)	0.509	0.000	3.341(0.580–22.824)	0.199
Cardiac involved	0.000	4.225(1.090–16.383)	0.001	0.000	5.370(1.910–15.472)	0.021
Gastrointestinal involved	0.204			0.584		
ILD	0.155			0.984		
Velcro crackles	0.000	2.918(1.340–6.354)	0.007	0.003	7.249(0.961–54.687)	0.055
PH	0.000	1.006(0.353–2.870)	0.991	0.016	1.079(0.250–4.656)	0.919
Hemoglobin(g/L)	0.672			0.541		
ANA	0.000	1.608(0.389–10.368)	0.334	0.289		
Scl-70	0.327	3.608(1.189–10.368)	0.000	0.216	3.569(1.333–10.869)	0.002
ACA	0.057			0.108		
Elevated Creatine kinase	0.858			0.696		
Elevated Myoglobin	0.000	2.313(0.620–8.630)	0.212	0.004	3.686(0.795–11.997)	0.075
Elevated BNP	0.000	2.600(1.070–8.776)	0.024	0.010	2.135(1.293–9.564)	0.044
Positive Coombs test	0.001	0.472(0.054–4.089)	0.495	0.000	0.608(0.149–3.986)	0.336
Elevated Cr	0.000	5.076(0.633–26.315)	0.201	0.000	7.504(0.872–18.676)	0.180
Elevated BUN	0.000	1.354(0.439–4.170)	0.598	0.000	3.208(0.949–10.986)	0.236
Elevated IgG	0.025	2.147(0.599–7.691)	0.240	0.284		
Decreased PaO2	0.000	2.229(0.738–6.733)	0.155	0.000	1.426(0.355–5.726)	0.617
Elevated CRP	0.000	2.356(0.728–7.626)	0.153	0.004	2.740(0.262–22.681)	0.400
Elevated ESR	0.000	1.191(0.328–4.322)	0.791	0.021	2.392(0.291–8.663)	0.417
Elevated RF	0.132			0.172		

## Discussion

In 1957, Erasmus [16] reported that gold miners exposed to silica had a higher incidence of SSc than did the general population (2/1000 vs 0.35/1000). Since then, various studies have also found the correlation between silicon exposure and SSc, but the results differ [2, 4–7]. Because of the small number of cases, there is a paucity of clinical literature regarding silicon dioxide and SSc, and even less regarding silicosis. We collected silicosis patients hospitalized in Guangxi Workers’ Hospital and the People's Hospital of Guangxi Zhuang Autonomous Region in the past 20 years, including 72 patients with SSc, which is the largest number of cases nationally and internationally so far.

Our study found that the age of onset of SSc in patients with silicosis was lower than that of patients without silicosis, which was inconsistent with international results [6, 7, 17]. We believe this is because most of the patients we included were miners who experiences high labor intensity. Silicosis, or dust exposure, accelerates the development of systemic sclerosis [8, 9]. Ferric [17] and Dedecker [6] failed to find the age difference between the two groups because they chose SSc patients with a history of exposure to silica, and there was a choice bias in those studies. However, Rochalf [7] only included nine cases, eight of which were diagnosed with silicosis; these low numbers could be attributed to age differences.

In this study, there was only one female in the group of 72 patients with SSc combined with silicosis. Compared with SSc patients without silicosis, the gender composition ratio was significantly different. There were more men in the SSc silicosis group because there were more men who had been exposed to silica for a long time. Mora et al. [2] also believe that occupational exposure to silica can significantly increase the risk of SSc in men, mainly because the men are more likely to be exposed. The results of studies varied regarding whether gender affected the condition of SSc patients exposed to silica. A case–control study showed that gender did not affect the incidence and severity of SSc [18, 19]. A recent study in France found that men with occupational exposure to silica had a higher risk of SSc (OR = 9.63) [20]. The same group analyzed 142 SSc patients and found that silica exposure was associated with diffuse SSc, ILD, systemic microvascular disease, and cancer. In the multivariate analysis, male sex was considered an important risk factor for SSc associated with silica exposure (OR = 19) [21].

Our study found that patients with SSc combined with silicosis had more ILD, more Velcro crackles, and lower partial pressure of oxygen, and the proportion of TB was significantly increased. Multivariate analysis showed that silicosis was an independent risk factor for death, and survival analysis also suggested that the survival rate of SSc patients with silicosis decreased. Marie et al. [21] found that ILD is often the first manifestation of SSc in exposed patients. These findings highlight the need for close monitoring of ILD in patients exposed to silica. The lung was the organ with high morbidity in the case–control study of SSc and silica. This may be affected by SSc, but we believe that silica is a key factor. Both silicosis and SSc-ILD are classified as interstitial lung diseases. The high-kV imaging signs of silicosis are mainly round or irregular small shadows. HRCT shows diffuse or scattered small shadows with clear edges, focused in the upper middle lung fields, or widely distributed irregular line shadows [22]. HRCT findings of SSc-ILD were characterized by nonspecific interstitial pneumonia (NSIP) and usual interstitial pneumonia (UIP) [23]. Sometimes, it is difficult to distinguish the components of ILD secondary to SSc or silicosis from clinical, radiologic, and even pathological perspectives, and this requires a decision from a multidisciplinary team comprising rheumatologists, respiratory doctors, radiologists, and pathologists [24].

Our study found that the SSc group with silicosis had more patients with cardiac involvement, and we also observed a significantly higher cumulative incidence of cardiac-related deaths in the SSc with silicosis group. Multivariate analysis also found that cardiac involvement was an independent risk factor for death. From the constituent ratio of cardiac involvement, the incidence of myocardial involvement and tricuspid regurgitation was higher in the SSc with silicosis group. We believe that this is the result of the combined effect of SSc itself and secondary lesions caused by silicosis. Compared with other studies on cardiac involvement in SSc patients with silicosis, Rocha et al. [7] only compared two groups with no difference in pulmonary arterial hypertension, and Marie et al. [21] found that patients exposed to silica had a lower median LVEF, after excluding confounding factors that might alter cardiac function, such as smoking habits and cardiovascular risk. Our patients with SSc complicated with silicosis had a higher incidence of PH. Comprehensive analysis of these patients revealed that silicosis patients also have severe lung lesions. We believe that this group includes both SSc-related PH, and pulmonary disease-related PH.

Previous studies have only focused on the clinical characteristics of patients. In this study, we analyzed the differences in treatment between the two groups for the first time. The results showed that the SSc group without silicosis was more likely to be administered high dose hormone impulsion therapy, adequate hormone therapy, and CTX, while patients with silicosis were more likely to be administered moderate hormone therapy, low-dose hormone therapy, and MTX. Currently, there are no guidelines for the treatment of SSc with silicosis. We believe that the physicians were more conservative when they treated SSc patients with silicosis with the use of hormones and immunosuppressants. Those patients in the SSc with silicosis group are prone to co-infection, including general bacterial infection and specific pathogens such as TB, and, in order to reduce the infection, doctors consciously reduce the dosage of hormones and immunosuppressive agents. Most of the 22 SSc patients without silicosis were treated with high dose hormones before 2015 (18 cases, 82%). In recent years, the use of high dose hormones in SSc has been controversial because of its possible relationship with SRC. However, it is generally believed that moderate to large doses of hormone therapy should be avoided for SSc patients as far as possible [25]. In our study, 15 cases of renal failure occurred in the SSc without silicosis group, among which five cases were treated with high dose hormone impulsion therapy. It was not clear whether the cause of renal failure was the disease itself or the effect of drugs. However, after 2015, we gradually reduced the use of high dose hormone therapy.

In this study, survival analysis showed that the prognosis was worse in the SSc group with silicosis, and that the main causes of death and irreversible organ damage were respiratory and cardiac failure, while in the SSc without silicosis group, the causes were primarily renal failure and hand/foot gangrene. Rocha [7] found that six of the nine exposed patients died of pulmonary complications during follow-up. Freire [26] found that the survival rate of SSc patients exposed to silica was lower than that of patients without silica exposure (p = 0.023), which was consistent with our results. The prognoses of the two groups in this study are different, which is also related to the inconsistency of the baseline between the two groups. To reduce bias from confounding factors between groups, PSM was performed. Matching covariates consisted of gender, age. SSc with silicosis was related with a higher overall mortality (HR = 2.817, 95% CI = 1.196–10.764, p = 0.014) after PSM. In our study, COX analysis showed that cardiac involvement was an independent risk factor for all-cause death. Cardiac involvement included pericardial effusion, myocardial involvement, tricuspid regurgitation and coronary artery involvement, among which pericardial effusion and tricuspid regurgitation had the highest incidence. Our study found that the incidence of pericardial effusion was high in the SSc combined with silicosis group, which may be related to the immune status [27, 28], but its specific mechanism still needs further study. We also found that the incidence of tricuspid valve involvement was significantly increased in the SSc combined with silicosis group. The possible causes included the underlying inflammatory burden, immune system activation [29], and co-existing lung disease.

We believe that the death and poor prognosis in the SSc with silicosis group are mainly related to cardiac involvement. In order to improve the prognosis of SSc patients with silicosis, it is necessary to focus on monitoring cardiac disease, actively treating silicosis, and possibly comorbid TB and pneumonia, weighing the advantages and disadvantages of hormone use, reducing the use of high dose hormones, and selecting appropriate immunosuppressants.

Nevertheless, this study has shortcomings. First, the number of patients was small. In the follow-up research, the sample size should be expanded, and the follow-up time needs to be extended in order to obtain more valuable clinical research results. Secondly, theoretically, the imaging appearance of silicosis and SSc-ILD is different. But sometimes it is difficult to distinguish the components of ILD secondary to SSc or silicosis. Many patients in our study had severe lung lesions, and silicosis and SSc-ILD were mixed together, especially in the middle and lower lung lesions, making it difficult to distinguish them.

## Conclusions

Studying the role of the environment in SSc will help to identify the possible pathogenic factors of disease. Evidence increasingly indicates that environmental factors play an important role in the regulation of epigenetic determinants, leading to the occurrence and development of SSc. In conclusion, all patients with SSc should be systematically screened for occupational exposure at the time of diagnosis, because identifying occupational toxic substances and interrupting their exposure may improve the prognosis of SSc. For individuals with occupational exposure, close observation of the symptoms of SSc, early diagnosis, and interruption of exposure may also improve the prognosis. Cardiac involvement has a significant role in the mortality of patients with SSc. Early identification of patients with cardiac involvement is essential. The advantages and disadvantages of available treatment options such as hormones and immunosuppressants have to be weighed, with emphasis on the patients medical status.

## Data Availability

Data and material are available from the corresponding author upon reasonable request.

## References

[1] Denton CP, Khanna D (2017). Systemic sclerosis. Lancet.

[2] Mora GF (2009). Systemic sclerosis: environmental factors. J Rheumatol.

[3] Ranque B, Mouthon L (2010). Geoepidemiology of systemic sclerosis. Autoimmun Rev.

[4] Dospinescu P, Jones GT, Basu N (2013). Environmental risk factors in systemic sclerosis. Curr Opin Rheumatol.

[5] Makol A, Reilly MJ, Rosenman KD (2011). Prevalence of connective tissue disease in silicosis (1985–2006)-a report from the state of Michigan surveillance system for silicosis. Am J Ind Med.

[6] De Decker E, Vanthuyne M, Blockmans D (2018). High prevalence of occupational exposure to solvents or silica in male systemic sclerosis patients: a Belgian cohort analysis. Clin Rheumatol.

[7] Rocha LF, Luppino Assad AP, Marangoni RG (2016). Systemic sclerosis and silica exposure: a rare association in a large Brazilian cohort. Rheumatol Int.

[8] Eleftheriadis T, Pissas G, Zarogiannis S, Liakopoulos V, Stefanidis I (2019). Crystalline silica activates the T-cell and the B-cell antigen receptor complexes and induces T-cell and B-cell proliferation. Autoimmunity.

[9] Pollard KM (2016). Silica, Silicosis, and Autoimmunity. Front Immunol.

[10] Preliminary criteria for the classification of systemic sclerosis (scleroderma) (1980). Subcommittee for scleroderma criteria of the American Rheumatism Association Diagnostic and Therapeutic Criteria Committee. Arthritis Rheum.

[11] LeRoy EC, Black C, Fleischmajer R (1988). Scleroderma (systemic sclerosis): classification, subsets and pathogenesis. J Rheumatol.

[12] Muszyńska-Graca M, Dąbkowska B, Brewczyński PZ (2016) Guidelines for the use of the international classification of radiographs of pneumoconioses of the international labour office (ILO): substantial changes in the currrent edition. Medycyna Pracy 67(6):833–837. 10.13075/mp.5893.00493 10.13075/mp.5893.00493 28005090

[13] Firestein G, Budd R, Gabriel SE (2012). Kelley's Textbook of Rheumatology.

[14] Lewinsohn DM, Leonard MK, LoBue PA (2017). Official American Thoracic Society/Infectious Diseases Society of America/Centers for Disease Control and Prevention Clinical Practice Guidelines: Diagnosis of Tuberculosis in Adults and Children. Clin Infect Dis.

[15] Boyd CM, Xue QL, Simpson CF, Guralnik JM, Fried LP (2005). Frailty, hospitalization, and progression of disability in a cohort of disabled older women. Am J Med.

[16] Erasmus LD (1957). Scleroderma in goldminers on the Witwatersrand with particular reference to pulmonary manifestations. S Afr J Lab Clin Med.

[17] Ferri C, Artoni E, Sighinolfi GL (2018). High serum levels of silica nanoparticles in systemic sclerosis patients with occupational exposure: Possible pathogenetic role in disease phenotypes. Semin Arthritis Rheum.

[18] Rosenman KD, Moore-Fuller M, Reilly MJ (1999). Connective tissue disease and silicosis. Am J Ind Med.

[19] Englert H, Small-McMahon J, Davis K (2000). Male systemic sclerosis and occupational silica exposure-a population-based study. Aust N Z J Med.

[20] Marie I, Gehanno JF, Bubenheim M (2014). Prospective study to evaluate the association between systemic sclerosis and occupational exposure and review of the literature. Autoimmun Rev.

[21] Marie I, Menard JF, Duval-Modeste AB (2015). Association of occupational exposure with features of systemic sclerosis. J Am Acad Dermatol.

[22] Champlin J, Edwards R, Pipavath S (2016). Imaging of Occupational Lung Disease. Radiol Clin North Am.

[23] Distler O, Assassi S, Cottin V et al (2020) Predictors of progression in systemic sclerosis patients with interstitial lung disease. Eur Respir J 55(5):1902026. 10.1183/13993003.02026-2019 10.1183/13993003.02026-2019 PMC7236865 32079645

[24] Wallis A, Spinks K (2015). The diagnosis and management of interstitial lung diseases. BMJ.

[25] Blagojevic J, Legendre P, Matucci-Cerinic M (2019). Is there today a place for corticosteroids in the treatment of scleroderma?. Autoimmun Rev.

[26] Freire M, Alonso M, Rivera A (2015). Clinical peculiarities of patients with scleroderma exposed to silica: A systematic review of the literature. Semin Arthritis Rheum.

[27] García MA, Alarcón GS, Boggio G (2014). Grupo Latino Americano de Estudio del Lupus Eritematoso (GLADEL). Primary cardiac disease in systemic lupus erythematosus patients: protective and risk factors–data from a multi-ethnic Latin American cohort. Rheumatology (Oxford)..

[28] Sugiura A, Funabashi N, Ozawa K (2016). Immunological and inflammatory processes in systemic autoimmune disease may not only cause pericardium inflammation, but may also cause mitral valve deterioration and left ventricular wall thickening. Int J Cardiol.

[29] Bissell LA, Anderson M, Burgess M (2017). Consensus best practice pathway of the UK Systemic Sclerosis Study group: management of cardiac disease in systemic sclerosis. Rheumatology (Oxford).

